# SNP4OrphanSpecies: A bioinformatics pipeline to isolate molecular markers for studying genetic diversity of orphan species

**DOI:** 10.3897/BDJ.10.e85587

**Published:** 2022-08-24

**Authors:** Benjamin Penaud, Benoit Laurent, Marine Milhes, Camille Noüs, François Ehrenmann, Cyril Dutech

**Affiliations:** 1 BIOGECO, INRAE, Univ. Bordeaux, 33610 Cestas, France BIOGECO, INRAE, Univ. Bordeaux 33610 Cestas France; 2 INRAE, US 1426, GeT-PlaGe, Genotoul, Castanet-Tolosan, France INRAE, US 1426, GeT-PlaGe, Genotoul Castanet-Tolosan France; 3 Laboratoire Cogitamus, Bordeaux, France Laboratoire Cogitamus Bordeaux France

**Keywords:** amplicon, biological invasion, forest diseases and pests, single-copy genes, whole-genome sequencing

## Abstract

**Background:**

For several decades, an increase in disease or pest emergences due to anthropogenic introduction or environmental changes has been recorded. This increase leads to serious threats to the genetic and species diversity of numerous ecosystems. Many of these events involve species with poor or no genomic resources (called here "orphan species"). This lack of resources is a serious limitation to our understanding of the origin of emergent populations, their ability to adapt to new environments and to predict future consequences to biodiversity. Analyses of genetic diversity are an efficient method to obtain this information rapidly, but require available polymorphic genetic markers.

**New information:**

We developed a generic bioinformatics pipeline to rapidly isolate such markers with the goal for the pipeline to be applied in studies of invasive taxa from different taxonomic groups, with a special focus on forest fungal pathogens and insect pests. This pipeline is based on: 1) an automated de novo genome assembly obtained from shotgun whole genome sequencing using paired-end Illumina technology; 2) the isolation of single-copy genes conserved in species related to the studied emergent organisms; 3) primer development for multiplexed short sequences obtained from these conserved genes. Previous studies have shown that intronic regions of these conserved genes generally contain several single nucleotide polymorphisms within species. The pipeline's functionality was evaluated with sequenced genomes of five invasive or expanding pathogen and pest species in Europe (*Armillariaostoyae* (Romagn.) Herink 1973, *Bursaphelenchusxylophilus* Steiner & Buhrer 1934, *Sphaeropsissapinea* (fr.) Dicko & B. Sutton 1980, *Erysiphealphitoides* (Griffon & Maubl.) U. Braun & S. Takam. 2000, *Thaumetopoeapityocampa* Denis & Schiffermüller, 1775). We successfully isolated several pools of one hundred short gene regions for each assembled genome, which can be amplified in multiplex. The bioinformatics pipeline is user-friendly and requires little computational resources. This easy-to-set-up and run method for genetic marker identification will be useful for numerous laboratories studying biological invasions, but with limited resources and expertise in bioinformatics.

## Introduction

Pest and disease emergences are an important threat to the biodiversity and functioning of world ecosystems (Fisher et al. 2012) and human well-being (Diagne et al. 2021). A dramatic increase in these events has been recorded for several decades (Anderson et al. 2004, Fisher et al. 2012, Santini et al. 2012, Lips 2016, Rohr et al. 2019). Specifically, in the context of invasions or recent expansions of pests and parasites, there is an urgent need to identify methods for avoiding, stopping or, at least, reducing their spread and deleterious effects on ecosystems, species and genetic diversity (Filipe et al. 2012, Gonthier et al. 2014, Hall et al. 2016) . For many of the emerging pests and pathogens, however, taxonomic and biological knowledge is insufficient, with no or little information about their geographical origin, routes of colonisation and ability to adapt to newly-colonised environments and environmental change, for example, induced by human activities (Lavergne and Molofsky 2007, Fraimout et al. 2017, Gross et al. 2021).

Evolutionary and demographic inferences may be obtained from population genetic analyses (Estoup and Guillemaud 2010, Beichman et al. 2018). For emerging species that are generally little studied before causing significant effects on ecosystems or native species, we need to develop new genetic markers because of no or few published genetic resources. This development is sometimes challenging because, to obtain correct genetic estimates, markers should only amplify DNA of the species under investigation. Population genetic studies are often based on samples collected in the field. This sampling may cause, especially for micro-organisms, DNA contamination due to host or other associated micro-organisms in the samples (Ballenghien et al. 2017) or possible mistakes of identification due to the occurrence of cryptic species in the sampled area (i.e. species apparently identical morphologically, but incapable of producing hybrids) (Queloz et al. 2011, Mapondera et al. 2012, Gross et al. 2021). In some cases, the situation is even more challenging when hybridisation may occur amongst sympatric species or between invasive and native species (Sillo et al. 2015, Soghigian et al. 2020). However, the genotyping of multiple polymorphic markers may sometimes allow the identification of the different genetic lineages and their potential hybrids (Altermann et al. 2014, López-Vinyallonga et al. 2015). As late as the first decade of the 21^st^ century, microsatellite markers (also called simple sequence repeats, SSR) were extensively used for population genetic analyses (Selkoe and Toonen 2006). With the emergence of next-generation sequencing (NGS) technologies, single nucleotide polymorphism markers (SNP) have been increasingly used for population genetic studies.

Out of the methods used for SNP genotyping, genome-wide sequencing (GWS) allows us to obtain thousands of markers by using high-throughput DNA sequencing on parts or whole genomes studied (Elshire et al. 2011). These methods can be powerful because they generate a huge quantity of genetic information. However, they may be costly in time and money, since they generally need good DNA quality for all the samples analysed, high sequencing coverage to identify duplicated genomic regions and several computational steps for removing putative false positive SNPs (Ribeiro et al. 2015). All these requirements can be difficult for some species having a genome with numerous repetitive elements, for species for which extraction of good quality DNA is not easy to obtain (Dutech et al. 2020) or for research teams without bioinformatics expertise. In addition, the large genetic information generated by GWS methods may be beyond the need of only a few genetic markers to first estimate reproductive mode, gene flow, spatial structure or origin of the expansion of emerging species (Peccoud et al. 2008, Brodde et al. 2019). Alternatively to GWS, several studies have shown that it is generally possible to isolate a few SNPs within introns of single-copy genes conserved in the genus or the family of targeted species (Feau et al. 2011, Ilves and López-Fernández 2014) or in ultraconserved elements (UCEs) (Blaimer et al. 2015, Bossert and Danforth 2018). The method developed by Feau et al. (2011) has been successfully applied to several fungal pathogens and it also allowed us to investigate population genetic structure, gene flow and reproductive mode (Dutech et al. 2017, Tsykun et al. 2017, Dutech et al. 2020). Based on detecting a few single-copy genes in the genomes, this method has yielded less than fifty unlinked SNPs per study and it has not been automated to reduce analysis time.

Therefore, the objective of the present software was to develop an automatic bioinformatics pipeline usable for a large number of species, especially focusing on emerging forest pest and pathogen species that are often orphan species (i.e. with no or poorly published genomic resources). The pipeline hereafter called “SNP4OrphanSpecies” is designed to be easily installed and used by biologists and based on limited genomic resources (i.e. one single shotgun whole genome sequencing) in order to provide useful genetic markers for assignments to genetic lineages, identification of the origin of invading or expanding populations and estimates of population diversity and structure.

## Project description

### Title

SNP4OrphanSpecies

### Design description

The method to isolate single-copy genes for orphan species is based on an automated de novo genome assembly without the step of manual curation, using sequence data generated by paired-end Illumina sequencing technology. The assembly quality is checked by looking at some summary statistics (i.e. genome size, degree of assembly fragmentation, completeness of the genome). For isolating hundreds of SNPs, we focused on the single-copy genes conserved in genomes at a given taxonomic level (i.e. genus, family or order). The focus on these conserved genes allows us: 1) to control for the taxonomic assignment of the analysed genomic regions, 2) to remove duplicated genes in the genome which can produce possible false positive SNPs, 3) to isolate several SNPs generally present in the introns of these genes and 4) to yield several pools of pairs of primers for amplification of around 400 bp sequences which can be amplified together in one multiplex (100 sequences per pool). By automation of these steps, the method decreases the time of genomic analysis, while it selects DNA sequences specific to the studied species (discarding sequences due to, for example, laboratory or field DNA contamination; Ballenghien et al. 2017). Based on the first polymorphic sequences obtained from this method in Feau et al. (2011) and Dutech et al. (2016), we expect that the isolated sequences with intron regions are polymorphic within species and valuable candidates for future SNPs detection.

**Figure 1.**
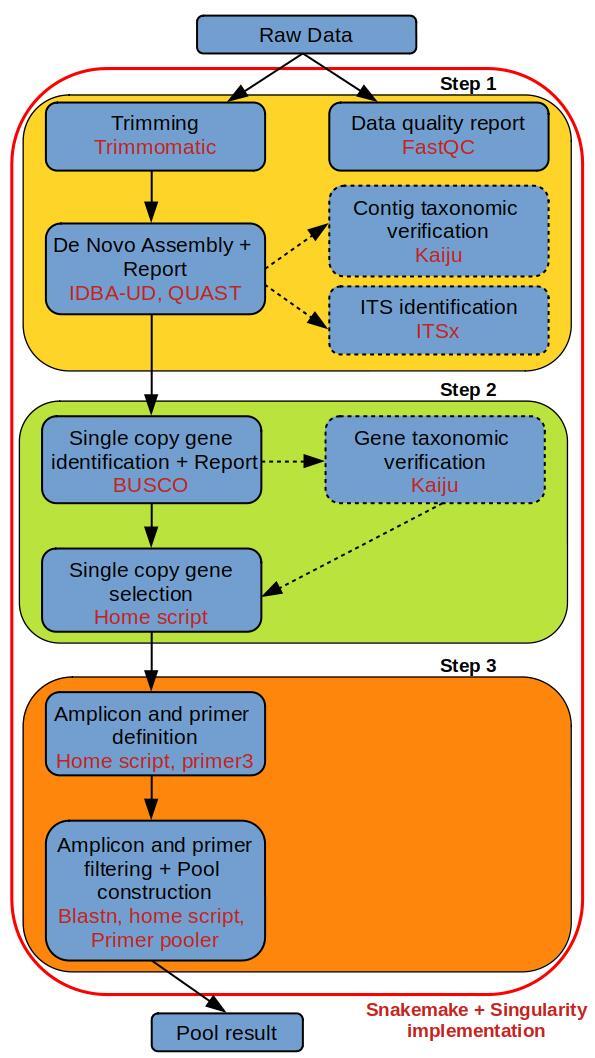
Figure 1: steps of the SNP4OrphanSpecies pipeline.

The basic pipeline steps are illustrated in Fig. 1. The first step of the pipeline is the whole-genome de novo assembly using paired-end short reads obtained from Illumina technology. Although not tested in this study, minimum coverage of 10X is recommended for correcting sequencing error, and probably 20X minimum to obtain a correct de novo assembly (Jiang et al. 2019). This step starts with a quality analysis of the raw data using FastQC v.0.11.9 (https://www.bioinformatics.babraham.ac.uk/projects/fastqc/). Reads are trimmed by using a sliding window and filtered using a minimum length with the software trimmomatic v.0.39 (Bolger et al. 2014). The parameters for trimming are defined in the parameter file of the pipeline (Snakemake_Config_SNP4OrphanSpecies.yaml). Then a de novo assembly is performed using IDBA-UD v.1.1.3 (Peng et al. 2010). A basic statistics report is then generated on the final assembly using Quast v.5.0.2 (Gurevich et al. 2013). For fungi, bacteria and viruses only, detection for DNA contamination can be performed by an automatic assignation of the assembled contigs, using Kaiju v.1.7.4 (Menzel et al. 2016). Additionally, for fungal species only, isolation of the internal transcribed spacer (ITS) can be performed from the de novo genome assembly with ITSx v.1.1b (Bengtsson-Palme et al. 2013).

The second step of this pipeline evaluates the completeness of the assembly and identifies the genes which will be used to isolate short sequences (400 bp, hereafter called “amplicon”). In this objective, BUSCO v.4.1.4 (Seppey et al. 2019) runs on the de novo assembly obtained in the previous step to identify single-copy genes conserved at the taxonomic level defined in the the configuration file (Snakemake_Config_SNP4OrphanSpecies.yaml). The more narrowly the taxonomic information is defined, the more specific are the isolated markers. Then, only complete and not duplicate genes are kept for the definition of amplicons. Optionally, a taxonomic assignment of the selected genes can be done using Kaiju. Genes assigned to taxon other than the taxon set in the configuration file are removed from the final selection. This verification step requires large disc spaces (ideally 125 GB for the nr_euk database) and is possible only for fungi, bacteria and viruses.

The last step of the pipeline is the isolation of amplicons to be amplified in pools. For this step, the amplicons are chosen to encompass at least one intron in the sequence. For each amplicon, a pair of primers is designed using a home-made Perl script integrating the programme Primer3 (v.2.5 Koressaar and Remm 2007), with stringent parameters favouring the possibility to be amplified jointly in a single multiplex PCR. All the designed primers are subjected to BLASTn (v.2.10, Altschul et al. 1990) against the de novo genome assembly to test for the specificity of the targeted sequences. Each pair of primers for which one of the two primers was found in at least two copies in the genome, was removed. One pair of primers is finally randomly selected per BUSCO gene to amplify a maximum of physically unlinked sequences. The validated primers are dispatched in several pools for which the primer dimer formation compatibility during a multiplex DNA amplification is tested in silico, using Primer Pooler (v.1.71, Brown et al. 2017).

## Web location (URIs)

Homepage: https://doi.org/10.15454/GWKRKY

## Technical specification

Programming language: snakemake v.6 or later and singularity v.3 or later.

Operational system: Linux; Hardware requirements (Minimum): 32 GB of RAM, 1 CPU

Interface language: Command line

## Repository

Type: Dataverse

Browse URI: https://data.inrae.fr/

Location: https://doi.org/10.15454/GWKRKY

## Usage licence

### Usage licence

Creative Commons Public Domain Waiver (CC-Zero)

## Implementation

### Implements specification

Keeping in mind biologist users, we implemented this pipeline with Snakemake (Köster and Rahmann 2012) and Singularity (Kurtzer et al. 2017). These softwares allow us to organise the whole bioinformatics workflow within a container, with all software and libraries needed and the automatic achievement of each step of the analysis from the initial input data. This pipeline is easy to install, easy to use and can be run on all Linux machines, including high-performance clusters. The pipeline, its associated notice and parameter files can be downloaded from the Portail Data INRAE (https://doi.org/10.15454/GWKRKY).

For running the pipeline, users only must: 1) produce a paired-end Illumina whole-genome sequencing of the species of interest and 2) set parameters (i.e. taxonomy, filtering, number of amplicons), in the file “Snakemake_Config_SNP4OrphanSpecies.yaml”. A README available on https://doi.org/10.15454/GWKRKY gives more details about these different steps and parameters.

### Audience

We consider that the time of bioinformatics analyses to isolate and to develop new markers is seriously reduced thanks to this pipeline, easily installed on a personal computer, without the need to access the internet after this setting. Then, this method is especially dedicated to research teams, governmental agencies or organisations, which have limited human and financial resources. With the short sequences provided by this pipeline, the possibility of obtaining the first genetic information on recently emerging populations without the high cost of genome sequencing should help to identify the origin of emergence and the risk of adaptation to new ecosystems and define the best practices to manage new disease or pest species.

## Additional information

### Pipeline assessment

We assessed the performance of the pipeline in a new de novo genome assembly of *Diplodiasapinea* isolate CBS117911. *Diplodiasapinea* or *Sphaeropsissapinea* (fr.) Dicko & B. Sutton 1980 is a worldwide emergent fungal pathogen infecting many host trees, especially pine species (Brodde et al. 2019). A genomic library was constructed for this isolate using the Illumina TruSeq Nano DNA kit, following the company procedure. A total of 10,544,224 raw 150 bp paired-end reads were generated by an Illumina HiSeq3000 sequencer at the Get-Plage Genotoul facility (INRAE, France). In addition, we used this pipeline for analysing four other invasive species from different phyla, for which the genome assembly has been already published (Table 1). *Erysiphealphitoides* (Griffon & Maubl.) U. Braun & S. Takam. 2000, (Ascomycota) infects many host plants worldwide and was likely introduced to Europe at the beginning of the 20^th^ century (Gross et al. 2021). Genomic resources have recently been published for this obligate biotroph species (i.e. non-culturable on axenic media), for which DNA contamination was detected in the genome assembly (Dutech et al. 2020). *Armillariaostoyae* (Romagn.) Herink 1973 (Basidiomycota) is distributed throughout the Northern Hemisphere, infecting numerous conifer species, causing large loss of wood (Heinzelmann et al. 2019) and is associated with some expanding populations in planted forests, as assumed in south-western France (Labbé et al. 2017). The whole genome sequencing, published by Sipos et al. (2017), was used for this study. *Thaumetopoeapityocampa* Denis & Schiffermüller 1775 (Lepidoptera) is expanding in Europe due to climatic changes and causes significant defoliation in pine plantations and human health concerns (Battisti and Larsson 2015). The genome sequencing used to assess the pipeline has been published by Gschloessl et al. (2018). *Bursaphelenchusxylophilus* Steiner & Buhrer 1934, a pine wood nematode, infects several pine species and was introduced from its native North America area to Asia and Europe, where it causes dramatic mortality in forests of the invaded areas (Vicente et al. 2011). The genome used for this study was published by Dayi et al. (2020).

After filtering and trimming Illumina raw reads, new de novo genome assemblies were produced by the pipeline for each species (details of each assembly are given in Table 1). These genome assemblies were strongly fragmented with small L50 and large N50 values (Table 1). As expected for contaminated DNA extraction (Dutech et al. 2020), the *E.alphitoides* genome assembly was one of the most fragmented genomes together with *T.pityocampa*. It confirmed the originally published surprisingly large estimate of the genome size for a powdery mildew species (317 Mb vs. less than 110 Mb for other published powdery mildew genome assemblies; Frantzeskakis et al. 2019). For fungal species, identifying the ITS1 sequences using the Kaiju nr_euk database confirmed that at least a part of the genome assembly may be assigned to the expected genus for each sequenced species (Suppl. material 1). The software ITSx used for this identification detected several ITS1 haplotypes in the *E.alphitoides* genome, which is congruent with the detection of several contigs of the genome assigned to different phyla or fungal families (Suppl. material 2). Between 98.6% (*D.sapinea*) and 42% (*T.pityocampa*) of conserved single-copy genes listed in the Busco database were isolated from the genome assemblies (Table 2). For the analysed fungal species and using Kaiju, a variable proportion of genes was actually identified as different from the targeted genus, leading to the removal of between 70% (*E.alphitoides*) and 0.6% (*A.ostoyae*) of the initial list of single-copy genes. In the last steps of the analysis, the pipeline defined in each species between 20,991 (*A.ostoyae*) and 1,829 (*E.alphitoides*) short 400 bp sequences (i.e. amplicons), encompassing at least one intron region. The design of the primer pairs for DNA amplification for each amplicon (only one per gene) and the control for their potential duplication in the genomes yielded a final set of between 614 (*E.alphitoides*) and 3,426 (*A.ostoyae*) primer pairs (Table 2). All these primer pairs are pooled in five pools for multiple DNA amplification. Depending on the final number of designed primer pairs, the redundancy rates of primer pairs amongst the primer pools for each species varied between 19% (*A.ostoyae*) and 57.4% (*E.alphitoides*) (Table 2). This redundancy can be manually optimised amongst pools when rates are too high. Primer Pooler was not designed to build several pools of primers simultaneously and it may be useful to sequentially remove the pairs of primers used in the first pools to build the next pools.

### Perspectives for evolutionary genetic studies of non-model invasive and emergent insect and pathogen species

The analysis of genomes from different phyla suggested that the method can be used for a large number of invasive or emergent insect and pathogen species for which genetic markers are searched. Some limitations may occur for large genomes (i.e. several hundreds Mb), since the assembly, even without any curation steps, requires a minimum of computation resources. For example, the analysis of the *T.pityocampa* genome for which the size was estimated to be more than 500 Mb, generated 268 GB (only 134 GB for the trimmed fastaQ files) and took more than 18 hrs on a Linux cluster using 20 CPUs. For such a large genome, it could be interesting to assess the method with a reduced whole-genome sequencing (i.e. a lower sequencing coverage or randomly amplified genome). Based on our results obtained from the highly fragmented and contaminated *E.alphitoides* genome assembly, we speculate that several hundreds of the conserved single-copy genes can be generally isolated by the present method, even from a low-quality or partial genome assembly. Another limitation would be the use of contaminated genome assemblies which may be quite frequent in whole-genome sequencing (Ballenghien et al. 2017), especially for not easily cultivated micro-organisms. The smallest number of validated sequences was obtained for *E.alphitoides* for which such contamination was assumed (Dutech et al. 2020) and confirmed in this study. When it involves genetically related species (for example, between fungal species), such contamination may be difficult to identify and remove from the genomic data, because of the genetic similarity amongst the sequences. Using the Kaiju nr_euk database to assign the identified genes is then useful to detect this DNA contamination and discard the sequences with the incorrect taxonomic assignment. For the identification of conserved single-copy genes, a taxonomic determination is a sensitive parameter. An incorrect taxonomic identification at the genus level or higher of the emergent species may lead us to discard several BUSCO genes present in the genome assembly, but too different from the chosen reference genome. Conversely, if BUSCO genes are searched at a taxonomical level that includes the emergent species and a contaminating related species, some BUSCO genes non-specific to the emergent species may be selected despite the control performed by Kaiju. To avoid the selection of these unspecific genes, the isolated BUSCO genes can be evaluated again before the step of primers design. This evaluation can be done using the fasta files of the isolated genes (available in the outputs of the pipeline) and a phylogenetic reconstruction for each gene by extracting BUSCO genes of the taxonomical group studied from a database (for example, Mycocosm; Grigoriev et al. 2013) and the use of one of the numerous published phylogenetic software (see, for example, Dereeper et al. 2008).

Two strategies can then be developed after the isolation of these amplicons. The first one would be sequencing hundreds of samples using one of the designed pools and next-generation sequencers. The combination of SNPs identified in each amplicon can be treated as microhaplotypes (i.e. multi-SNP loci), potentially giving more power for population genetic analyses than analysing independent bi-allelic SNP loci (Baetscher et al. 2017). Microhaplotypes defined on short sequences, as those isolated by this pipeline, may be well adapted to identify fine population genetic structures, especially for field samples for which the quality of DNA extraction is often poor (Morin et al. 2021) or for assignment of samples to populations and estimates of population admixture (McKinney et al. 2017). In a preliminary PCR test on 47 samples of *D.sapinea*, the majority of the 100 pairs of primers tested in multiplex allowed us to obtain, on average, more than ten sequencing reads per sample and per amplicon. This last result is encouraging new tests on other pools of primers and on other orphan species. A second option would be the genotyping of selected SNPs (one per locus), combined in different pools (i.e. plex), on several hundreds of samples, using, for example, Mass-ARRAY technology by Sequenom (Chancerel et al. 2013, Dutech et al. 2017). Resequencing about ten genomes or partial genomes can first identify variations within each amplicon and then, several SNP-plex can be designed for genotyping hundreds of samples (Dutech et al. 2016). Although the haplotypic information is missed in this case and this strategy of genotyping may introduce ascertainment bias due to the choice of SNPs (Albrechtsen et al. 2010), it allows for genetically characterising many samples from different geographical regions, a central objective when the genetic origin of emergent populations is investigated.

We are aware that SNPs isolated from conserved genes may be under selection. It may seriously affect the inferences of demographic dynamics of populations and should be carefully considered if historical scenarios are tested (Beichman et al. 2018). A first study having selected these conserved genes in *Armillaria* sp., detected two out of 20 tested (Dutech et al. 2016). Notwithstanding this potential bias, we argue that for the first estimation of population genetic structures or identification of the genetic origin of the emergent populations, the methodology presented here remains efficient. No significant difference in the estimates of genetic structures has been observed when comparing SSR and SNP loci isolated using this method in several European populations of *A.cepistipes* (Tsykun et al. 2017). Basic statistics can also identify loci under selection (Vitalis 2003). Furthermore, because these loci are chosen in conserved genes at a given taxonomic level, the designed primers can theoretically amplify every species of this level. This robustness for DNA amplification is useful for investigating genetic differentiation amongst closely-related species and their hybrids (Altermann et al. 2014). Some loci, such as SSR loci, are sometimes difficult to transfer even within the same genus (Dutech et al. 2007) and may produce many null alleles and missing data. By contrast, the short sequences obtained by this pipeline would be especially relevant for studying genetic diversity and differentiation amongst closely-related species, because of the expected good repeatability of DNA amplification, the standardisation of genotyping amongst species, experiments or laboratories, as well as the assessment of the sequence orthology within genomes. They may be an efficient alternative to WGS methods for studying genetically related species or comparing or combining, genetic studies produced by different studies or laboratories (see Harvey et al. 2016 for details).

## Supplementary Material

3B316472-B302-56B1-9D5C-2C0B5D1EB5DB 10.3897/BDJ.10.e85587.suppl1 Supplementary material 1 ITS detected in the three fungal genome assemblies Data type Table File: oo_672329.odt https://binary.pensoft.net/file/672329 Penaud, Benjamin; Laurent, Benoît; Marine Milhes; Camille Noûs; François Erhemann; Dutech, Cyril.

0BDFBF5A-2A9E-592E-8516-39A187D4E7D6 10.3897/BDJ.10.e85587.suppl2 Supplementary material 2 Proportions of the genome assemblies assigned to different taxa using Kaiju Data type Figures File: oo_672331.pdf https://binary.pensoft.net/file/672331 Penaud, Benjamin; Laurent, Benoît; Marine Milhes; Camille Noûs; François Erhemann; Dutech, Cyril.

## Figures and Tables

**Table 1.** Description of the genome assemblies obtained for the five tested species. Data into brackets are from the original publication.

**Species**	***Diplodiasapinea***	***Erysiphealphitoides***	***Armillariaostoyae***	***Thaumetopoeapityocampa***	***Bursaphelenchusxylophilus***
class	Dothideomycetes	Leotiomycetes	Agaricomycocetes	Insecta	Secermentea
order	Botryosphaeriales	Erysiphales	Agaricales	Lepidoptera	Aphelenchida
family	Botryosphaeriaceae	Erysiphaceae	Physalacriaceae	Notodontidae	Parasitaphelenchidae
					
Reference	This study (reference genome: van der Nest et al. 2014)	Dutech et al. (2020)	Sipos et al. (2017)	Sipos et al. (2017)	Dayi et al. (2020)
Sequencing	Illumina Hiseq3000	Illumina Hiseq2000	Illumina Hiseq2000	Illumina Hiseq2000	Illumina Hiseq2000
Strain	CBS117911	MS_42D	C18	PE300i -PE600i	Ka4C1
Number of Reads	10,544,224	369,262,818	116,828,130	462,786,916	58,326,120
Number of Reads Used to construct the assembly	9,044,726	313,340,218	103,921,206	381,071,842	55,197,190
Total length	37,650,182 (36.97 Mb)	316,911,737 (308.4 Mb)	57,720,627 (60.9 Mb)	536,111,310 (537 Mb)	70,264,222 (74.6 Mb)
Nbcontigs > 500 bp	1,793 (2,194)	131,582 (555,289)	7,119 (106)	289,399 (68,292)	10,373 (5,527)
Nbcontigs > 1000 bp	1,387	79,253	4,666	185,303	7,823
Nbcontigs > 50000 bp	200	68	215	1	76
Largest contig	324,688	102,030	563,590	63,395	148,994
GC(%)	56.71	49.73	48.32	38.08	40.38
N50 (kb)	48.5 (37.7)	3.4 (1.7)	34.3 (2800)	2.3 (163.6)	15.1 (949)
L50 (number)	218 (NA)	17,657 (NA)	371 (9)	67,374 (728)	1,341 (22)

**Table 2.** Summary of the genes and primers isolated by SNP4Orphanspecies pipeline for the five tested species. * one per gene and not duplicated in the genome.

**Species**	***Diplodiasapinea***	***Erysiphealphitoides***	***Armillariaostoyae***	***Thaumetopoeapityocampa***	***Bursaphelenchusxylophilus***
Nb of Busco genes	3,786	3,234	3,870	5,286	3,131
Nb of Complete single-copy	3,733	2,353	3,787	2,219	2,068
Nb with the validated genus	3,557	987	3,765	NA	NA
Nb of defined amplicons	6,962	1,829	20,991	3,163	13,256
Nb of genes with amplicons	2,760	685	3,438	887	1,955
Nb of pairs of primers	6,095	1,408	20,617	1,916	10,938
Nb of conserved pairs of primers*	2,570	614	3,426	672	1,928
% gene duplication in pools	20.8	57.4	19	52.8	23

## References

[B7929029] Albrechtsen Anders, Nielsen Finn Cilius, Nielsen Rasmus (2010). Ascertainment biases in SNP chips affect measures of population divergence.. Molecular biology and evolution.

[B7831007] Altermann Susanne, Leavitt Steven D., Goward Trevor, Nelsen Matthew P., Lumbsch H. Thorsten (2014). How do you solve a problem like *Letharia*? A new look at cryptic species in lichen-forming fungi using Bayesian clustering and SNPs from multilocus sequence data. PLOS One.

[B7940106] Altschul S F, Gish W, Miller W, Myers E W, Lipman D J (1990). Basic local alignment search tool.. Journal of molecular biology.

[B7830314] Anderson Pamela K., Cunningham Andrew A., Patel Nikkita G., Morales Francisco J., Epstein Paul R., Daszak Peter (2004). Emerging infectious diseases of plants: pathogen pollution, climate change and agrotechnology drivers. Trends in Ecology & Evolution.

[B7832049] Baetscher Diana S., Clemento Anthony J., Ng Thomas C., Anderson Eric C., Garza John C. (2017). Microhaplotypes provide increased power from short‐read DNA sequences for relationship inference. Molecular Ecology Resources.

[B7830909] Ballenghien Marion, Faivre Nicolas, Galtier Nicolas (2017). Patterns of cross-contamination in a multispecies population genomic project: detection, quantification, impact, and solutions. BMC Biology.

[B7831961] Battisti A, Larsson S, Bjorkman C, Niemela P (2015). Climate Change and Insect Pests.

[B7830325] Beichman Annabel C., Huerta-Sanchez Emilia, Lohmueller Kirk E. (2018). Using genomic data to infer historic population dynamics of nonmodel organisms. Annual Review of Ecology, Evolution, and Systematics.

[B7831829] Bengtsson-Palme Johan, Ryberg Martin, Hartmann Martin, Branco Sara, Wang Zheng, Godhe Anna, De Wit Pierre, Sánchez-García Marisol, Ebersberger Ingo, de Sousa Filipe, Amend Anthony S., Jumpponen Ari, Unterseher Martin, Kristiansson Erik, Abarenkov Kessy, Bertrand Y. J. K., Sanli Kemal, Eriksson K. M, Vik Unni, Veldre Vilmar, Nilsson R. Henrik (2013). Improved software detection and extraction of ITS1 and ITS2 from ribosomal ITS sequences of fungi and other eukaryotes for analysis of environmental sequencing data. Methods in Ecology and Evolution.

[B7831599] Blaimer Bonnie B., Brady Seán G., Schultz Ted R., Lloyd Michael W., Fisher Brian L., Ward Philip S. (2015). Phylogenomic methods outperform traditional multi-locus approaches in resolving deep evolutionary history: a case study of formicine ants. BMC Evolutionary Biology.

[B7831735] Bolger Anthony M., Lohse Marc, Usadel Bjoern (2014). Trimmomatic: a flexible trimmer for Illumina sequence data. Bioinformatics.

[B7831610] Bossert Silas, Danforth Bryan N. (2018). On the universality of target‐enrichment baits for phylogenomic research. Methods in Ecology and Evolution.

[B7830351] Brodde Laura, Adamson Kalev, Julio Camarero J., Castaño Carles, Drenkhan Rein, Lehtijärvi Asko, Luchi Nicola, Migliorini Duccio, Sánchez-Miranda Ángela, Stenlid Jan, Özdağ Şule, Oliva Jonàs (2019). Diplodia tip blight on its way to the north: Drivers of disease emergence in northern Europe. Frontiers in Plant Science.

[B7830368] Brown Silas S., Chen Yun-Wen, Wang Ming, Clipson Alexandra, Ochoa Eguzkine, Du Ming-Qing (2017). PrimerPooler: automated primer pooling to prepare library for targeted sequencing. Biology Methods and Protocols.

[B7830379] Chancerel Emilie, Lamy J - B, Lesur Isabelle, Noirot Céline, Klopp Christophe, Ehrenmann François, Boury Christophe, Provost Grégoire Le, Label Philippe, Lalanne Céline, Léger Valérie, Salin Franck, Gion Jean-Marc, Plomion Christophe (2013). High-density linkage mapping in a pine tree reveals a genomic region associated with inbreeding depression and provides clues to the extent and distribution of meiotic recombination. BMC Biology.

[B7830398] Dayi Mehmet, Sun Simo, Maeda Yasunobu, Tanaka Ryusei, Yoshida Akemi, Tsai Isheng Jason, Kikuchi Taisei (2020). Nearly complete genome assembly of the pinewood nematode *Bursaphelenchusxylophilus* strain Ka4C1. Microbiology Resource Announcements.

[B7832032] Dereeper A., Guignon V., Blanc G., Audic S., Buffet S., Chevenet F., Dufayard J. - F., Guindon S., Lefort V., Lescot M., Claverie J. - M., Gascuel O. (2008). Phylogeny.fr: robust phylogenetic analysis for the non-specialist. Nucleic Acids Research.

[B7830300] Diagne Christophe, Leroy Boris, Vaissière Anne-Charlotte, Gozlan Rodolphe E., Roiz David, Jarić Ivan, Salles Jean-Michel, Bradshaw Corey J. A., Courchamp Franck (2021). High and rising economic costs of biological invasions worldwide. Nature.

[B7832117] Dutech Cyril, Enjalbert Jérome, Fournier Elisabeth, Delmotte François, Barrès Benoit, Carlier Jean, Tharreau Didier, Giraud Tatiana (2007). Challenges of microsatellite isolation in fungi. Fungal Genetics and Biology.

[B7830410] Dutech C., Prospero S., Heinzelmann R., Fabreguettes O., Feau N. (2016). Rapid identification of polymorphic sequences in non-model fungal species: the PHYLORPH method tested in *Armillaria* species. Forest Pathology.

[B7830420] Dutech Cyril, Labbé Frédéric, Capdevielle Xavier, Lung-Escarmant Brigitte (2017). Genetic analysis reveals efficient sexual spore dispersal at a fine spatial scale in *Armillariaostoyae*, the causal agent of root-rot disease in conifers. Fungal Biology.

[B7830438] Dutech C., Feau N., Lesur I., Ehrenmann F., Letellier T., Li B., Mouden C., Guichoux E., Desprez-Loustau M. L., Gross A. (2020). An easy and robust method for isolation and validation of single-nucleotide polymorphic markers from a first *Erysiphealphitoides* draft genome. Mycological Progress.

[B7830453] Elshire Robert J., Glaubitz Jeffrey C., Sun Qi, Poland Jesse A., Kawamoto Ken, Buckler Edward S., Mitchell Sharon E. (2011). A robust, simple Genotyping-by-Sequencing (GBS) approach for high diversity species. PLOS One.

[B7830888] Estoup Arnaud, Guillemaud Thomas (2010). Reconstructing routes of invasion using genetic data: why, how and so what?. Molecular Ecology.

[B7830465] Feau Nicolas, Decourcelle Thibaut, Husson Claude, Desprez-Loustau Marie-Laure, Dutech Cyril (2011). Finding single copy genes out of sequenced genomes for multilocus phylogenetics in non-model fungi. PLOS One.

[B7830709] Filipe João A. N., Cobb Richard C., Meentemeyer Ross K., Lee Christopher A., Valachovic Yana S., Cook Alex R., Rizzo David M., Gilligan Christopher A. (2012). Landscape epidemiology and control of pathogens with cryptic and long-distance dispersal: Sudden oak death in northern Californian forests. PLOS Computational Biology.

[B7830288] Fisher Matthew C., Henk Daniel. A., Briggs Cheryl J., Brownstein John S., Madoff Lawrence C., McCraw Sarah L., Gurr Sarah J. (2012). Emerging fungal threats to animal, plant and ecosystem health. Nature.

[B7830751] Fraimout Antoine, Debat Vincent, Fellous Simon, Hufbauer Ruth A., Foucaud Julien, Pudlo Pierre, Marin Jean-Michel, Price Donald K., Cattel Julien, Chen Xiao, Deprá Marindia, François Duyck Pierre, Guedot Christelle, Kenis Marc, Kimura Masahito T., Loeb Gregory, Loiseau Anne, Martinez-Sañudo Isabel, Pascual Marta, Polihronakis Richmond Maxi, Shearer Peter, Singh Nadia, Tamura Koichiro, Xuéreb Anne, Zhang Jinping, Estoup Arnaud (2017). Deciphering the routes of invasion of *Drosophilasuzukii* by means of ABC random forest. Molecular Biology and Evolution.

[B7831995] Frantzeskakis Lamprinos, Németh Márk Z., Barsoum Mirna, Kusch Stefan, Kiss Levente, Takamatsu Susumu, Panstruga Ralph (2019). The *Parauncinulapolyspora* draft genome provides insights into patterns of gene erosion and genome expansion in powdery mildew fungi. mBio.

[B7830722] Gonthier P., Anselmi N., Capretti P., Bussotti F., Feducci M., Giordano L., Honorati T., Lione G., Luchi N., Michelozzi M., Paparatti B., Sillo F., Maria Vettraino A., Garbelotto M. (2014). An integrated approach to control the introduced forest pathogen *Heterobasidionirregulare* in Europe. Forestry.

[B7832013] Grigoriev Igor V., Nikitin Roman, Haridas Sajeet, Kuo Alan, Ohm Robin, Otillar Robert, Riley Robert, Salamov Asaf, Zhao Xueling, Korzeniewski Frank, Smirnova Tatyana, Nordberg Henrik, Dubchak Inna, Shabalov Igor (2013). MycoCosm portal: gearing up for 1000 fungal genomes. Nucleic Acids Research.

[B7830475] Gross Andrin, Petitcollin Célia, Dutech Cyril, Ly Bayo, Massot Marie, Faivre d’Arcier Julie, Dubois Laure, Saint-Jean Gilles, Desprez-Loustau Marie-Laure (2021). Hidden invasion and niche contraction revealed by herbaria specimens in the fungal complex causing oak powdery mildew in Europe. Biological Invasions.

[B7830489] Gschloessl B., Dorkeld F., Berges H., Beydon G., Bouchez O., Branco M., Bretaudeau A., Burban C., Dubois E., Gauthier P., Lhuillier E., Nichols J., Nidelet S., Rocha S., Sauné L., Streiff R., Gautier M., Kerdelhué C. (2018). Draft genome and reference transcriptomic resources for the urticating pine defoliator *Thaumetopoeapityocampa* (Lepidoptera: Notodontidae). Molecular Ecology Resources.

[B7831798] Gurevich Alexey, Saveliev Vladislav, Vyahhi Nikolay, Tesler Glenn (2013). QUAST: quality assessment tool for genome assemblies. Bioinformatics.

[B7830741] Hall R. J., Castilla G., White J. C., Cooke B. J., Skakun R. S. (2016). Remote sensing of forest pest damage: a review and lessons learned from a Canadian perspective. The Canadian Entomologist.

[B7832130] Harvey Michael G., Smith Brian Tilston, Glenn Travis C., Faircloth Brant C., Brumfield Robb T. (2016). Sequence capture versus restriction site associated DNA sequencing for shallow systematics. Systematic Biology.

[B7830512] Heinzelmann Renate, Dutech Cyril, Tsykun Tetyana, Labbé Frédéric, Soularue Jean-Paul, Prospero Simone (2019). Latest advances and future perspectives in *Armillaria* research. Canadian Journal of Plant Pathology.

[B7831536] Ilves Katriina L., López-Fernández Hernán (2014). A targeted next-generation sequencing toolkit for exon-based cichlid phylogenomics. Molecular Ecology Resources.

[B7831685] Jiang Yifan, Jiang Yao, Wang Sheng, Zhang Qin, Ding Xiangdong (2019). Optimal sequencing depth design for whole genome re-sequencing in pigs. BMC Bioinformatics.

[B7831855] Koressaar T., Remm M. (2007). Enhancements and modifications of primer design program Primer3. Bioinformatics.

[B7830531] Köster J., Rahmann S. (2012). Snakemake—a scalable bioinformatics workflow engine. Bioinformatics.

[B7830548] Kurtzer Gregory M., Sochat Vanessa, Bauer Michael W. (2017). Singularity: Scientific containers for mobility of compute. PLOS One.

[B7830557] Labbé F, Fontaine M C, Robin C, Dutech C (2017). Genetic signatures of variation in population size in a native fungal pathogen after the recent massive plantation of its host tree. Heredity.

[B7830782] Lavergne Sébastien, Molofsky Jane (2007). Increased genetic variation and evolutionary potential drive the success of an invasive grass. Proceedings of the National Academy of Sciences.

[B7830574] Lips Karen R. (2016). Overview of chytrid emergence and impacts on amphibians. Philosophical Transactions of the Royal Society B: Biological Sciences.

[B7831017] López-Vinyallonga Sara, López-Pujol Jordi, Constantinidis Theophanis, Susanna Alfonso, Garcia-Jacas Núria (2015). Mountains and refuges: Genetic structure and evolutionary history in closely related, endemic *Centaurea* in continental Greece. Molecular Phylogenetics and Evolution.

[B7830919] Mapondera Tendai S, Burgess Treena, Matsuki Mamoru, Oberprieler Rolf G (2012). Identification and molecular phylogenetics of the cryptic species of the *Gonipterusscutellatus* complex (Coleoptera: Curculionidae: Gonipterini). Australian Journal of Entomology.

[B7832080] McKinney Garrett J., Seeb James E., Seeb Lisa W. (2017). Managing mixed-stock fisheries: genotyping multi-SNP haplotypes increases power for genetic stock identification. Canadian Journal of Fisheries and Aquatic Sciences.

[B7831820] Menzel Peter, Ng Kim Lee, Krogh Anders (2016). Fast and sensitive taxonomic classification for metagenomics with Kaiju. Nature Communications.

[B7832059] Morin Phillip A., Forester Brenna R., Forney Karin A., Crossman Carla A., Hancock‐Hanser Brittany L., Robertson Kelly M., Barrett‐Lennard Lance G., Baird Robin W., Calambokidis John, Gearin Pat, Hanson M. Bradley, Schumacher Cassie, Harkins Timothy, Fontaine Michael C., Taylor Barbara L., Parsons Kim M. (2021). Population structure in a continuously distributed coastal marine species, the harbor porpoise, based on microhaplotypes derived from poor‐quality samples. Molecular Ecology.

[B7831456] Peccoud J., Figueroa C. C., Silva A. X., Ramirez C. C., Mieuzet L., Bonhomme J., Stoeckel S., Plantegenest M., Simon J. - C. (2008). Host range expansion of an introduced insect pest through multiple colonizations of specialized clones. Molecular Ecology.

[B7831789] Peng Yu, Leung Henry C. M., Yiu S. M., Chin Francis Y. L. (2010). IDBA – A practical lterative de Bruijn Graph de novo assembler. Lecture Notes in Computer Science.

[B7830928] Queloz V., Grünig C. R., Berndt R., Kowalski T., Sieber T. N., Holdenrieder O. (2011). Cryptic speciation in *Hymenoscyphusalbidus*. Forest Pathology.

[B7831323] Ribeiro Antonio, Golicz Agnieszka, Hackett Christine Anne, Milne Iain, Stephen Gordon, Marshall David, Flavell Andrew J., Bayer Micha (2015). An investigation of causes of false positive single nucleotide polymorphisms using simulated reads from a small eukaryote genome. BMC Bioinformatics.

[B7830590] Rohr Jason R., Barrett Christopher B., Civitello David J., Craft Meggan E., Delius Bryan, DeLeo Giulio A., Hudson Peter J., Jouanard Nicolas, Nguyen Karena H., Ostfeld Richard S., Remais Justin V., Riveau Gilles, Sokolow Susanne H., Tilman David (2019). Emerging human infectious diseases and the links to global food production. Nature Sustainability.

[B7830609] Santini A., Ghelardini L., Pace C., Desprez‐Loustau M. L., Capretti P., Chandelier A., Cech T., Chira D., Diamandis S., Gaitniekis T., Hantula J., Holdenrieder O., Jankovsky L., Jung T., Jurc D., Kirisits T., Kunca A., Lygis V., Malecka M., Marcais B., Schmitz S., Schumacher J., Solheim H., Solla A., Szabò I., Tsopelas P., Vannini A., Vettraino A. M., Webber J., Woodward S., Stenlid J. (2012). Biogeographical patterns and determinants of invasion by forest pathogens in Europe. New Phytologist.

[B7831045] Selkoe Kimberly A., Toonen Robert J. (2006). Microsatellites for ecologists: a practical guide to using and evaluating microsatellite markers. Ecology Letters.

[B7831811] Seppey Mathieu, Manni Mosè, Zdobnov Evgeny M. (2019). BUSCO: Assessing genome assembly and annotation completeness. Methods in Molecular Biology.

[B7830989] Sillo Fabiano, Garbelotto Matteo, Friedman Maria, Gonthier Paolo (2015). Comparative genomics of sibling fungal pathogenic taxa identifies adaptive evolution without divergence in pathogenicity genes or genomic structure. Genome Biology and Evolution.

[B7830645] Sipos György, Prasanna Arun N., Walter Mathias C., O’Connor Eoin, Bálint Balázs, Krizsán Krisztina, Kiss Brigitta, Hess Jaqueline, Varga Torda, Slot Jason, Riley Robert, Bóka Bettina, Rigling Daniel, Barry Kerrie, Lee Juna, Mihaltcheva Sirma, LaButti Kurt, Lipzen Anna, Waldron Rose, Moloney Nicola M., Sperisen Christoph, Kredics László, Vágvölgyi Csaba, Patrignani Andrea, Fitzpatrick David, Nagy István, Doyle Sean, Anderson James B., Grigoriev Igor V., Güldener Ulrich, Münsterkötter Martin, Nagy László G. (2017). Genome expansion and lineage-specific genetic innovations in the forest pathogenic fungi *Armillaria*. Nature Ecology & Evolution.

[B7830977] Soghigian John, Gloria‐Soria Andrea, Robert Vincent, Le Goff Gilbert, Failloux Anna‐Bella, Powell Jeffrey R. (2020). Genetic evidence for the origin of *Aedesaegypti*, the yellow fever mosquito, in the southwestern Indian Ocean. Molecular Ecology.

[B7830699] Tsykun T, Rellstab C, Dutech C, Sipos G, Prospero S (2017). Comparative assessment of SSR and SNP markers for inferring the population genetic structure of the common fungus *Armillariacepistipes*. Heredity.

[B7834577] van der Nest Magriet A., Bihon Wubetu, De Vos Lieschen, Naidoo Kershney, Roodt Danielle, Rubagotti Enrico, Slippers Bernard, Steenkamp Emma T., Wilken P. Markus, Wilson Andrea, Wingfield Michael J., Wingfield Brenda D. (2014). Draft genome sequences of *Diplodiasapinea*, *Ceratocystismanginecans*, and *Ceratocystismoniliformis*. IMA Fungus.

[B7831974] Vicente Cláudia, Espada Margarida, Vieira Paulo, Mota Manuel (2011). Pine wilt disease: a threat to European forestry. European Journal of Plant Pathology.

[B7832108] Vitalis R. (2003). DetSel 1.0: A computer program to detect markers responding to selection. Journal of Heredity.

