# Supplementary figures and images for: SNP4OrphanSpecies: A bioinformatics pipeline to isolate molecular markers for studying genetic diversity of orphan species
Source: Biodivers Data J. 2022 Aug 24;10:e85587. doi: 10.3897/BDJ.10.e85587 (PMC9848450; doi:10.3897/BDJ.10.e85587)

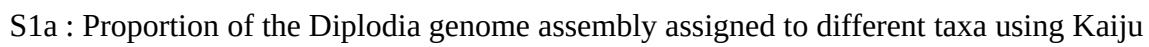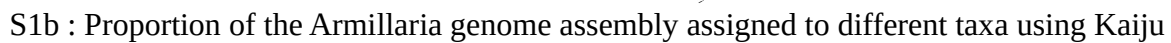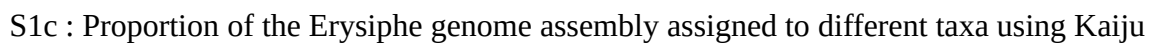

Supplement: Supplementary material 2 — Proportions of the genome assemblies assigned to different taxa using Kaiju [file bdj-10-e85587-s002.pdf]
