# Supplementary material for: SNP4OrphanSpecies: A bioinformatics pipeline to isolate molecular markers for studying genetic diversity of orphan species
Source: Biodivers Data J. 2022 Aug 24;10:e85587. doi: 10.3897/BDJ.10.e85587 (PMC9848450; doi:10.3897/BDJ.10.e85587)
Supplement: Supplementary file 3 [file bdj-10-e85587-s001.odt]

Table S1 : ITS detected in the three fungal genome assemblies

| Genome Assembly | Sequence | Length | Assignment | Phylum | Class | Coverage | Identity |
| --- | --- | --- | --- | --- | --- | --- | --- |
| Diplodia sapinea | ITS1_DS_1 | 174 | Diplodia sp. | Ascomycota | Dothideomycetes | 100 | 100 |
|  |  |  |  |  |  |  |  |
| Armillaria ostoyae | ITS1_AO_1 | 239 | Armillaria sp. | Basidiomycota | Agaricomycetes | 100 | 100 |
|  |  |  |  |  |  |  |  |
| Erysiphe alphitoides | ITS1_EA_1 | 950 | Thrips tabaci | Arthropoda | Insecta | 99 | 99 |
|  | ITS1_EA_2 | 220 | Erisyphe alphitoides | Ascomycota | Leotiomycetes | 100 | 100 |
|  | ITS1_EA_3 | 111 | Wallemia sebi / W. muriae | Basidiomycota | Wallemiomycetes | 100 | 100 |
|  | ITS1_EA_4 | 142 | Aspergillus sp. | Ascomycota | Eurotiomycetes | 100 | 100 |
|  | ITS1_EA_5 | 122 | Wallemia hedera | Basidiomycota | Wallemiomycetes | 100 | 99 |
|  | ITS1_EA_6 | 175 | Penicillium sp1. | Ascomycota | Eurotiomycetes | 100 | 100 |
|  | ITS1_EA_7 | 173 | Penicillium sp2. | Ascomycota | Eurotiomycetes | 100 | 100 |
